# Circulating tumor cells in breast cancer bone metastasis: mechanisms, clinical relevance, and future directions

**DOI:** 10.1038/s41420-025-02910-1

**Published:** 2025-12-10

**Authors:** Lingfeng Ma, Yufei Wang, Shuying Qiu, Min Shi, Wanfen Tang, Hangqi Hu, Chentao Li, Haiqi Lu, Xian Wang

**Affiliations:** 1 Department of Medical Oncology, Sir Run Run Shaw hospital, School of Medicine, Zhejiang University, Hangzhou, Zhejiang China; 2 Laboratory of Cancer Biology, Key Lab of Biotherapy in Zhejiang, Sir Run Run Shaw Hospital, School of Medicine, Zhejiang University, Hangzhou, Zhejiang China; 3 Department of Medical Oncology, Zhejiang Putuo Hospital, Zhoushan, Zhejiang Province China; 4 Department of Medical Oncology, Affiliated Jinhua Hospital, Zhejiang University School of Medicine, Jinhua, Zhejiang China; 5 Department of Pathology, Sir Run Run Shaw Hospital, Zhejiang University School of Medicine, Hangzhou, China

**Keywords:** Breast cancer, Tumour biomarkers, Bone metastases

## Abstract

Circulating tumor cells (CTCs) play a critical role in the metastatic cascade and have emerged as promising biomarkers for cancer diagnosis, prognosis, and therapeutic monitoring. In breast cancer, CTCs mediate bone metastasis through intricate interactions with the bone microenvironment, regulating process such as homing, dormancy, reactivation, and colonization. Advances in CTC detection and characterization have deepened our understanding of their physical and biological properties, yet significant technical and biological challenges remain. This review provides a comprehensive overview of the roles of CTCs in breast cancer bone metastasis and highlighting their clinical significance, current limitations, and future applications.

## Facts

1. Breast cancer–derived CTCs exhibit phenotypic plasticity, including epithelial-mesenchymal transition, subtype switching and cluster formation, which is associated with bone metastatic potential.
2. CTCs regulate dormancy, reactivation, and osteolytic progression in bone metastasis, positioning them as functional mediators of disease evolution.
3. Advances in CTC detection and molecular profiling enable minimally invasive monitoring of metastatic risk and therapeutic response, though technical and biological challenges limit clinical translation.

## Open questions

1. Which CTC subtypes can best reflect the spatial and temporal dynamics of breast cancer bone metastasis?
2. What molecular pathways govern the switch between dormancy and reactivation of disseminated tumor cells in bone?
3. How can CTC detection and characterization be standardized and translated into clinically actionable tools for bone metastasis monitoring?

## Introduction

According to the most recent global cancer statistics published by the International Agency for Research on Cancer (IARC), ~20 million new cancer cases and 9.7 million cancer-related deaths occurred worldwide in 2022. Breast cancer is the second most frequently diagnosed malignancy and the leading cause of cancer-related mortality among women globally [1]. Metastasis is the primary cause of breast cancer-related deaths, driving disease progression and therapeutic resistance.

Metastasis refers to the dissemination of cancer cells from the primary tumor to distant organs, with the skeleton being one of the most common metastatic sites in breast cancer. Among patients with advanced breast cancer, ~75% develop bone metastases, and nearly 70% of breast cancer-related deaths are associated with bone lesions [2]. Bone metastases can lead to skeletal-related events (SREs), including bone pain, pathological fractures, spinal cord compression, and malignant hypercalcemia [3], significantly diminishing patients’ quality of life [4]. Moreover, bone metastases often indicate an incurable disease stage and are commonly associated with increased resistance to systemic therapies [5]. Recent evidence also suggests that bone metastases may serve as a “secondary soil”, promoting the emergency of more aggressive metastatic “seeds” for future dissemination [6]. In clinical practice, the management of bone metastases requires a multimodal approach. Systemic anticancer therapies such as endocrine therapy, chemotherapy, HER2-targeted agents and CDK4/6 inhibitors remain the cornerstone of treatment, aiming to control systemic disease progression and improve survival. Bone-targeted agents like zoledronic acid and denosumab are commonly used to prevent skeletal-related events. Additionally, radiotherapy and, in selected cases, surgery play important roles in symptom relief and structural stabilization. However, current treatments are rarely curative, and therapeutic decisions often rely on imaging or clinical symptoms, which may not fully reflect the dynamic biological behavior of bone metastases. Therefore, timely and accurate detection of bone metastases and assessment of therapeutic responses are crucial to improve clinical outcomes.

In recent years, CTCs have emerged as a valuable indicator for elucidating metastatic pathways and tracking tumor evolution [7]. CTCs refer to tumor cells shed from primary or metastatic sites that enter the peripheral bloodstream [8]. Through a variety of active and passive adaptation mechanisms, CTCs can adapt dynamically to influence the bone microenvironment remotely, facilitating colonization, proliferation, and ultimately metastasis [9]. Accordingly, CTCs have emerged as promising minimally invasive biomarkers for detecting bone metastases, monitoring disease progression, and assessing therapeutic response [10–13]. While their potential relevance to bone metastasis is increasingly recognized, current evidence supporting their role in the early detection of bone involvement remains preliminary and requires validation in prospective studies [14–16].

This review synthesizes recent literature on the biological characteristics and clinical implications of CTCs in breast cancer bone metastasis, with a focus on their emerging utility as biomarkers across the metastatic cascade.

## Physical and biological hallmarks of CTC

In 1869, Ashworth firstly observed abnormal cells in the blood of a metastatic cancer patient resembling those of the primary tumor, laying the foundation for CTC research [17]. Subsequent studies revealed that CTCs can be detected in the bloodstream during early-stage disease. CTCs may circulate as individual cells or as clusters, with the latter showing significantly higher metastatic potential.

The mechanical and physical properties of CTCs are closely linked with their biological state and function, and tumor progression and metastasis, as well as enrichment technologies. The size of CTCs varies depending on the primary tumor type. In general, CTCs are larger than normal blood cells, with diameters typically ranging from 15 to 25 μm [18]. This difference facilitates the initial isolation of CTCs from blood components using size-based filtration techniques. In addition to size, tumor tissues are characteristically stiffer than their healthy counterparts, primarily due to alterations in the extracellular matrix (ECM). Increased tissue stiffness can induce changes in gene expression that promote cellular motility and mechanical plasticity [19]. Studies have shown that compared to normal cells, CTCs exhibit greater motility and deformability, which is indicative of their metastatic potential. From an electrical perspective, tumor cells display significantly higher membrane capacitance than normal blood cells, suggesting structural alterations in the cell membrane [20]. These changes can affect signal transduction and alter ion channel dynamics, potentially contributing to tumor development and progression. In the context of liquid biopsy, this electrical distinction has been exploited through microfluidic platforms that employing dielectrophoretic principles to selectively enrich CTCs. These systems offer advantages including label-free functionality, high sensitivity, and cell viability retention.

Beyond physical traits, CTCs possess critical biological hallmarks, among which surface molecular markers are particularly important. Since the vast majority of solid tumors originate from epithelial tissues, epithelial cell adhesion molecule (EpCAM) has become a key target for CTC enrichment and detection [15]. Clinical evidence suggests that EpCAM-positive CTCs play a significant role in prognostic assessment during both early and metastatic stages of breast cancer [21]. The semiautomated CellSearch^®^ platform (Janssen Diagnostics, Raritan, NJ, USA) isolates breast cancer CTCs using ferromagnetic beads coated in EpCAM and defines CTCs according to morphological characteristics, positive expression of cytokeratins and absence of the leukocyte marker CD45. This method has been recognized as the only technology validated in large-scale, multicenter clinical trials [7].

Clinically, based on the expression of hormone receptors: estrogen receptor (ER), progesterone receptor (PR) and HER2 (human epidermal growth factor receptor 2, ERBB2), breast cancer is classified into three major subtypes: Luminal, HER2-enriched and triple-negative breast cancer (TNBC) [22]. The Luminal subtype can be further divided into Luminal A and Luminal B according to the expression level of the proliferation marker Ki-67, based on its expression measured via immunohistochemistry. CTCs derived from different subtypes of breast cancer typically express receptor profiles similar to those of the corresponding primary tumors. However, several studies have reported discrepancies in receptor expression between metastatic lesions or corresponding CTCs and their matched primary tumors, indicating a phenomenon referred to as molecular subtype switching [6]. Such phenotypic changes have been closely associated with cancer progression, metastasis organotropism and therapeutic resistance development in breast cancer.

**Fig. 1:**
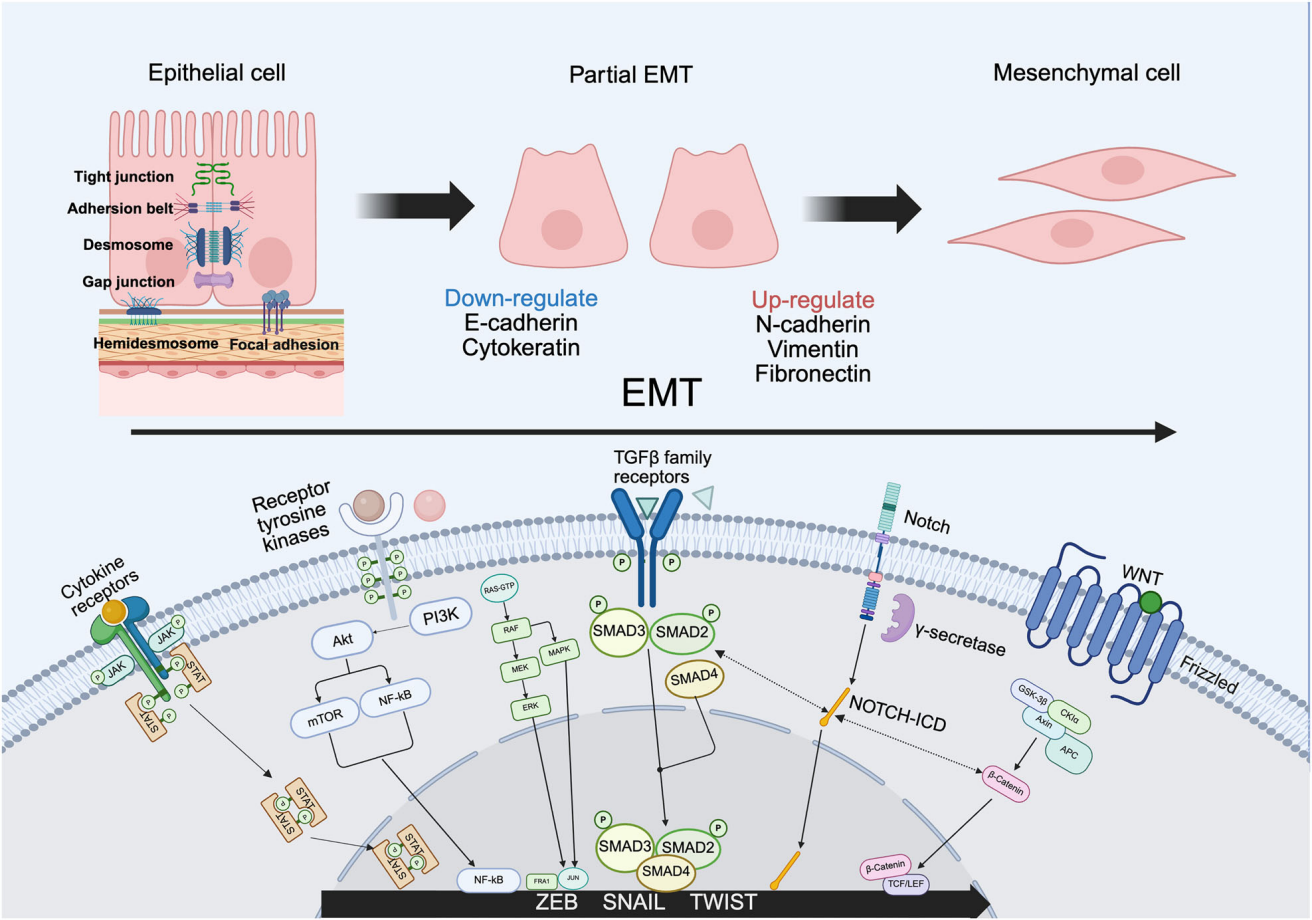
Overview of EMT process and associated signaling pathways in cancer. Tumor cells are interconnected through tight junctions, adhesion belts, desmosomes, and gap junctions, and anchored to the extracellular matrix via hemidesmosomes and focal adhesions. During EMT, cancer cells downregulate key epithelial markers, such as E-cadherin and cytokeratin, while upregulating mesenchymal markers, including N-cadherin, vimentin, and fibronectin. This transition is accompanied by morphological changes—from tightly packed, polygonal cells to loosely arranged, spindle-shaped cells, losing cell–cell adhesion, and enhancing ability to breach the basement membrane. Multiple signaling pathways, including TGF-β, Wnt, Notch, NF-κB, and various pro-inflammatory cytokines (e.g., IL-6, TNF-α), regulate the EMT process by activating core transcription factors such as SNAI, TWIST, and ZEB. These transcription factors repress epithelial gene expression and promote mesenchymal gene transcription, thereby driving phenotypic changes and endowing tumor cells with stem cell–like properties.

Phenotypic heterogeneity is a well-recognized feature of CTCs, largely attributed to their acquisition of distinct biological properties through epithelial-mesenchymal transition (EMT) [23]. EMT can be triggered by diverse tumor microenvironmental cues from the tumor microenvironment, including Wnt [24], Notch [25], TGF-β [26], cytokines [27], receptor tyrosine kinases [28], and hypoxia [29] as showed in Fig. 1. The induction of EMT is orchestrated by a set of core transcription factors (TFs), such as SNAI1/2, TWIST1/2, and ZEB1/2 [30]. These TFs exert pleiotropic effects by directly or indirectly repressing epithelial markers (e.g., E-cadherin [31], ZO-1 [32]) and promoting mesenchymal markers (e.g., Vimentin [33], N-cadherin [34]), leading to the loss of cell–cell and cell–matrix adhesion as well as apical–basal polarity, resulting enhanced migratory and invasive capacities [35]. Importantly, transcriptomic analyses have revealed that EMT is not a binary transition between epithelial and mesenchymal states [36]. Instead, tumor cells often exist along a spectrum of hybrid epithelial–mesenchymal phenotypes, many of which exhibit stem-like properties associated with increased tumorigenicity, propensity to metastasize and resistance to therapy [37]. However, because the EMT cascade largely involves intracellular changes, traditional detection strategies relying on surface markers are inadequate for isolating these subpopulations, underscoring the need for novel technologies to effectively capture and characterize EMT-phenotype CTCs.

In addition to single CTCs, CTC clusters, also known as circulating tumor microemboli (CTM), are frequently detected in the blood of patients with metastatic breast cancer. These clusters exhibit greater resistance to sheer stress and improved adhesion to vascular endothelial cells, enabling more efficient extravasation and colonization at distant sites [17, 38]. Recent studies suggest that CTC clusters possess significantly higher metastatic potential than solitary CTCs, with clusters of just two to three cells demonstrating markedly greater metastatic efficiency [39]. Tight junctions between cluster cells, along with their interactions with fibroblasts, platelets, and immune cells, are thought to enhance their metastatic capacity [40].

In summary, CTCs exhibit distinct characteristics in both physical and biological dimensions. These multi-level differences from normal tissues and primary tumors contribute significantly to various aspects of tumor progression, including initiation, metastasis, and therapeutic outcomes.

## Mechanism of CTC in bone metastasis

The metastasis of breast cancer is a complex yet coordinated process, and the biological mechanisms underlying its progression remain incompletely understood. Metastasis involves a series of sequential steps, beginning with the emergence of invasive, metastasis-competent cell clones within the primary tumor or pre-existing metastatic lesions that acquire the capacity to intravasate and enter the circulation. Once disseminated, these CTCs may extravasate, colonize permissive microenvironments, and ultimately give rise to secondary tumors.

**Fig. 2:**
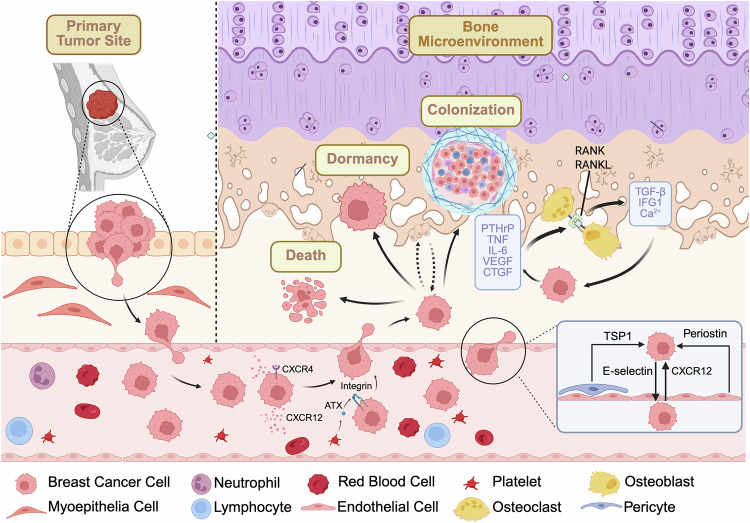
The metastatic cascade of breast cancer cells to the bone microenvironment. Breast cancer bone metastasis is a complex, multistep process. Tumor cells must first breach the basement membrane at the primary site and enter the circulation as circulating tumor cells (CTCs). In the bloodstream, CXCR12 and autotaxin (ATX) interact with receptors on CTCs, guiding their chemotactic migration toward the bone microenvironment. Subsequently, CTCs interact with endothelial cells and pericytes within the bone microvasculature, facilitating transendothelial migration and homing to the bone niche. Upon arrival, breast cancer CTCs can undergo one of three fates—apoptosis, dormancy, or colonization—without a fixed sequential order, and these states may coexist within the bone microenvironment. Dormant cells can be reactivated by factors secreted by osteoblasts. Successful bone metastasis formation is driven by a “vicious cycle,” wherein tumor-derived factors such as PTHrP, TNF, IL-6, VEGF, and CTGF promote RANKL–RANK interaction between osteoblasts and osteoclasts, leading to osteoclast activation and bone matrix degradation. This process releases TGF-β, IGF-1, and calcium ions, which in turn further stimulate breast cancer cell proliferation and secretory activity.

Among the various metastatic destinations, the bone represents a particularly common and clinically significant site in breast cancer. The propensity of CTCs to home to and colonize the bone is not a random event, but rather a result of sophisticated and dynamic interactions involving tumor cells, the bone niche, and systemic influences (Fig. 2). Elucidating how CTCs mediate and facilitate bone-specific metastasis is essential for understanding the organotropic nature of breast cancer dissemination and may reveal novel therapeutic targets to prevent or interrupt metastatic progression.

### Organotropsm

Although CTCs possess enhanced survival and dissemination capabilities, successful colonization requires permissive interactions with the bone microenvironment. To colonize the bone, CTCs rely on specialized mechanisms that facilitate homing, survival, and modulation of the local microenvironment. This organotropism aligns with the classic “seed and soil” theory, which posits that successful metastasis depends on the compatibility between CTCs (“seeds”) and the bone microenvironment (“soil”) [41].

A key mediator of this process is the CXCR4/CXCL12 chemokine axis. CTCs derived from breast cancer frequently exhibit high expression levels of the chemokine receptor CXCR4, while its ligand, CXCL12, is abundantly produced by mesenchymal stromal cells and pericytes in the bone niche [42]. The CXCL12-CXCR4 interaction generates chemotactic signals that guide CTCs toward the bone, reinforcing site-specific tropism [43]. Notably, bone tissue is inherently hypoxic, which induces stabilization and dimerization of hypoxia-inducible factor-1α (HIF-1α) [44]. The promoter region of the CXCR4 gene contains hypoxia-responsive elements (HREs), allowing dimerized HIF-1α to bind and enhance CXCR4 transcription [45]. This interaction creates a positive feedback loop that further amplifies CXCR4/CXCL12-mediated chemotaxis. Experimental studies have demonstrated that blocking this axis with neutralizing antibodies, or inhibiting downstream signaling components, significantly reduces the incidence of bone metastases [46].

In addition to chemotactic cues, the bone microenvironment actively contributes to metastatic colonization by secreting, or stimulating other cells to release, a range of prometastatic factors, such as milk fat globule-EGF factor 8 (MFGE8) [47], autotaxin (ATX) [48], RANK [49], integrin alpha-5 (ITGA5) [50], and sphingosine-1-phosphate receptor 1 (S1PR1) [51]. These molecules function as navigational cues or “metastatic nutrients” that further facilitate CTC adaptation and colonization within the bone niche.

## Homing and seeding

The bone marrow microenvironment composes two distinct niches: the perivascular niche and the endosteal niche. The perivascular niche, which includes vascular endothelial cells, pericytes, subendothelial stromal cells, and perivascular immune cells [52], serves as a critical entry point for circulating tumor cells (CTCs) to extravasate and engraft into the bone marrow [53]. Upon arrival, CTCs initially adhere to the vascular endothelium. This adhesion process is mediated by integrins [54], the glycoprotein complex GpIIb/IIIa [55], and P-selectin (SELP) [54, 56], which interact with their corresponding ligands on the CTCs surface. These interactions allow CTCs to evade immune surveillance and establish contact with endothelial cells, thereby facilitating transendothelial migration and entry into the perivascular niche [56], where they are termed disseminated tumor cells (DTCs).

Notably, this process is highly inefficient, with only ~0.2% of CTCs successfully colonizing distant sites [57]. Within the perivascular niche, DTCs preferentially localized to metaphyseal region enriched rich in type H vessels and induce endothelial sprouting to remodel the vasculature and establish a tumor-supportive microenvironment [58]. Following extravasation, chemotactic signals mediated by molecules such as CXCR4 and osteonectin (OCN), along with bone-derived factors including stromal cell-derived factor-1 (SDF-1/CXCL12), transforming growth factor-β1 (TGF-β1), and monocyte chemoattractant protein 1 (MCP-1, CCL2), guide DTCs deeper into the bone niche [59]. Final anchorage within the bone matrix is facilitated by adhesion molecules such as osteopontin (OPN), bone sialoprotein (BSP), CD44, collagen I (COL-1), and vascular cell adhesion molecule 1 (VCAM-1) [60].

## Dormancy and reactivation

After homing to the bone marrow, DTCs can enter a state of dormancy that may persist for several years until various stimuli trigger their reactivation [5]. Emerging evidence suggests that DTCs localize within bone marrow niches enriched in osteoblastic lineage cells, where they adopt a dormant phenotype. This process involves intricate interactions between tumor cells, osteolineage cells, and perivascular niche components. For instance, heterotypic adherens junctions (hAJs) formed by E-cadherin on DTCs and N-cadherin on osteoblasts can activate the mTOR signaling pathway, mediating tumor–niche communication [61]. In addition, several osteoblast-derived factors such as interleukin-6 (IL-6) [62], bone morphogenetic proteins (BMPs) [63], Wnt proteins [64], and TGF-β [65] have also been implicated in the maintenance of DTC dormancy. Endothelial cells also contribute by secreting thrombospondin-1 (TSP-1, THBS1), a matricellular protein known to maintain long-term tumor cell quiescence [66]. Dormant DTCs can be reactivated by stimuli such as bone remodeling and inflammation. In this context, VACM1 engages α4β1^+^osteoclast progenitors [67], prompting osteoclast-mediated niche remodeling disrupts dormancy-maintaining signals, enabling DTC reactivation, proliferation, and the formation of micrometastases [68].

## Colonization

The formation of micrometastases may further fuel osteoclastogenesis and reshape the bone microenvironment to favor tumor growth. During this process, breast cancer cells modulate osteoclastogenesis through paracrine signaling programs. Various cytokines and growth factors exert pleiotropic effects in stimulating osteoclast activity and promoting osteolytic metastasis. One of the key mediators is parathyroid hormone-related protein (PTHrP) [69], secreted by breast cancer cells and inducing osteoblasts to express receptor activator of NF-κΒ ligand (RANKL) [70]. RANKL binds to its receptor RANK on osteoclast precursors and metastatic cells, activating NF-κB signalin, promoting osteoclast differentiation and enhancing bone-resorbing activity [71]. During osteoclastic bone degradation, latent factors such as TGF-β, particularly TGF-β1, are released from the bone matrix in their active form ref. [72]. These factors further stimulate breast cancer cells to secrete additional PTHrP, thereby reinforcing a feed-forward loop known as the “vicious cycle” of osteolytic bone metastasis, ultimately resulting in extensive bone destruction [73]. In addition to the PTHrP–RANKL axis, other signaling pathways activated by VACM-1, including integrin [74], Notch [75], and RON [76], have also been implicated in amplifying this vicious cycle. As the metastatic lesion expands, tumor cells may become increasingly independent of niche-derived cues and potentially acquire the capacity to disseminate and colonize secondary metastatic sites.

In summary, CTC-driven bone metastasis is a multistep process. These steps are coordinated through dynamic interactions between tumor cells and the bone microenvironment, highlighting potential points of therapeutic intervention.

## Clinical role of CTC in bone metastasis of breast cancer

In recent years, CTCs have garnered significant attention as potential predictive biomarkers for tumor progression and metastasis. Table 1 summarizes clinical trials employing CTC-based strategies in breast cancer. Collectively, these studies demonstrate the feasibility of CTC-based monitoring and its prognostic relevance across both early and metastatic settings. Notably, several trials, including NCT01952054, NCT01129336, and NCT03070002, have investigated the potential of CTC enumeration to evaluate therapeutic response to denosumab or zoledronic acid in patients with bone metastases. However, no outcome data have yet been reported. Moreover, these trials focused solely on quantitative changes in CTC counts and did not explore phenotypic alterations or the biological significance of CTC clusters, which may provide deeper insights into treatment response and metastatic adaptation. In addition, in Table 1 we have included a brief conclusion explaining that many existing datasets allow retrospective or exploratory analyses correlating baseline CTC burden with subsequent metastasis events. However, these analyses vary in sample size and endpoint definition, making their conclusions necessarily preliminary, and most interventional trials do not provide organ-specific CTC analyses. Overall, most existing studies were not specifically designed to assess bone-related endpoints. These findings highlight both the promise and the present limitations of CTCs as clinical tools in breast cancer management. In the following section, we will explore the clinical utility and translational potential of CTCs in breast cancer bone metastasis.

**Table 1:** Representative clinical trials in breast cancer incorporating CTCs as outcome measures.

Trail ID	Disease type	Phase	CTC role	Detection method	Status	Results about CTCs	reference ID
NCT01548677	HER2-negative early breast cancer	II	Predict treatment response	CellSearch®	Completed	Using liquid biopsy to detect CTCs for identifying chemotherapy-resistant minimal residual disease and guiding targeted therapy in early breast cancer is feasible; pilot data reported in the trial publication describe successful CTC detection and provide rationale for CTC-guided interventional studies (no site-specific metastasis analysis reported).	PMID: 27289552
NCT03928210	Early Breast cancer	I	Predict treatment response	Parsortix™	Completed	CTC-driven treatment strategies in early breast cancer are feasible and safe, but the low rate of CTC-positive patients eligible for randomization limits the potential clinical impact, underscoring the need for improved CTC detection and selection methods to enhance future trial efficiency.	PMID: 39856336
NCT01619111	Hormone receptor–positive, HER2-negative early breast cancer	III	Predict treatment response	CellSearch®	Completed	In patients with hormone receptor–positive, HER2-negative early breast cancer at high risk of recurrence, CTC detection can help identify those who may benefit most from adjuvant ribociclib plus endocrine therapy, which significantly improves invasive disease-free survival.	PMID: 38175595
NCT00429182	Breast cancer	II	Predict treatment response	/	Completed	/	/
NCT01975142	HER2 negative metastatic breast cancer	II	Predict treatment response	CellSearch®	Completed	Studies screening HER2-amplified CTCs in HER2-negative metastatic patients identified a subset with HER2-amplified CTCs and explored HER2-directed therapy; clinical activity was limited in unselected populations. No specific site-targeted CTC analyses (e.g., bone vs liver) were reported. metastatic setting.	PMID: 31727113
NCT06067503	Breast cancer	II	Prognostic biomarker	VERSA (versatile exclusion-based rare sample analysis) platform	Recruiting	/	/
NCT02005770	Metastatic breast cancer	IV	Predict treatment response	CellSearch®	Completed	This trial and related work confirmed that CTC counts are prognostic in metastatic settings; the referenced publication addressed postoperative/anesthesia effects on postoperative CTC counts rather than organ-specific metastasis. No trial-level bone-specific CTC outcome reported.	PMID: 32568845
NCT00694252	Metastatic breast cancer	II	Predict treatment response	Lab-developed test	Completed	Monitoring HER2-positive CTCs in metastatic breast cancer demonstrates that lapatinib effectively reduces these cells regardless of the HER2 status of the primary tumor, highlighting the feasibility of using CTC molecular profiling to track treatment-induced changes during targeted therapy.	PMID: 26083256
NCT01129336	First or second line HER2-negative breast cancer, metastatic disease without bone metastasis	IV	Predict treatment response	/	Completed	/	/
NCT01185509	Breast cancer	II	Predict treatment response	Biocept CTC detection platform	Terminated	CTC analysis of patients with HER2-negative MBC identifies a subset with HER2-amplified CTCs. However, clinical activity of an HER2-directed regimen in this population was low. The functional significance of HER2-positive CTCs remains uncertain.	PMID: 34994617
NCT01349842	Third-line metastatic breast cancer	III	Prognostic biomarker	CellSearch®	Completed	CirCe01 prospective trial demonstrated that CTC enumeration and early CTC reduction during late-line chemotherapy are prognostic; dynamic CTC monitoring correlated with treatment outcome, supporting clinical validity of CTC kinetics in metastatic disease.	PMID: 25700777 PMID: 33473163
NCT03213041	HER2-negative breast cancer	II	Predict treatment response	/	Recruiting	/	/
NCT03070002	Hormone receptor–positive, HER2-negative breast cancer with bone metastasis	II	Predict treatment response	/	Terminated	/	/
NCT00820924	HER2-negative breast cancer	II	Predict treatment response	CellSearch^®^	Terminated	A subset of metastatic breast cancer patients with HER2-negative primary tumors harbor HER2-positive CTCs during disease progression, highlighting CTC profiling as a means to detect molecular shifts, although lapatinib demonstrated minimal clinical benefit in this population.	PMID: 23667487 PMID: 22476856
NCT02035813	HER2-negative and hormone-receptor positive metastatic breast cancer, postmenopausal female patients	II	Prognostic biomarker	/	Completed	/	/
NCT00382018	First-line breast cancer	III	Predict treatment response	CellSearch^®^	Completed	For patients with persistently increased CTCs after 21 days of first-line chemotherapy, early switching to an alternate cytotoxic therapy was not effective in prolonging OS. For this population, there is a need for more effective treatment than standard chemotherapy.	PMID: 24888818
NCT01952054	Breast cancer metastatic to bone	II	Predict treatment response	/	Terminated	/	/

This table summarizes representative clinical trials that employ CTCs as clinical or exploratory endpoints in breast cancer. Key parameters include disease setting, trial phase, current recruitment status, the specific role of CTCs, and the detection methodology. While several studies (e.g., NCT01952054, NCT01129336, NCT03070002) have evaluated the effects of bone-targeted therapies such as denosumab or zoledronic acid on CTC dynamics, no outcome data have yet been reported. Notably, these trials primarily focus on changes in CTC count rather than phenotypic alterations or CTC cluster biology, underscoring the need for future bone-specific and mechanistically oriented investigations. Collectively, these studies illustrate the feasibility of CTC-based monitoring but also highlight the current absence of prospective trials designed specifically to evaluate bone metastasis-related CTC dynamics.

CTCs offer potential advantages for providing real-time insights into disease progression and therapeutic response in patients with bone metastases, but their diagnostic specificity for bone involvement requires further validation. Numerous studies have demonstrated that elevated CTC counts are associated with an increased risk of metastasis, including an association with skeletal involvement in some retrospective and exploratory analyses [77]. In early-stage breast cancer patients, the presence of even a small number of CTCs in peripheral blood has been correlated with a higher likelihood of future metastatic relapse. For instance, a threshold of ≥5 CTCs per 7.5 mL of blood, as detected by the FDA-approved CellSearch^®^ system, has been widely used to stratify patients by risk, and its predictive value has been validated in several prospective trials [78]. Importantly, Longitudinal changes in CTC levels may, in some cases, precede radiographic progression and thus have potential to indicate occult skeletal disease; however, prospective validation is needed to confirm their sensitivity and specificity for early bone-metastasis detection [79]. Their accessibility through minimally invasive blood draws enables serial monitoring, which might be particularly valuable given the often indolent and radiographically occult nature of skeletal metastases.

Phenotypic heterogeneity of CTCs mirrors the spatial and temporal heterogeneity of cancer, reflecting tumor evolution under therapeutic pressure and highlighting the value of CTC phenotyping in capturing resistance mechanisms not evident in tissue biopsies. In breast cancer bone metastasis, CTCs often display molecular signatures indicative of organotropism and therapeutic response [80]. For instance, high CXCR4 expression facilitates bone marrow homing and has also been proposed as a therapeutic target through CXCR4/CXCL12 blockade in preclinical models [42, 46, 81]. Similarly, osteomimicry markers such as BSP [82], OPN [83], and RUNX2 [84, 85] enhance adhesion, survival, and drug resistance within osteogenic niches. And the identification of high trefoil factor 1(TFF1) expression in CTCs as a key feature of bone metastasis has been reported [13]. Subtype switching further contributes to treatment adaptation: loss of hormone receptor expression in HR⁺ CTCs correlates with poor prognosis under endocrine therapy [86], whereas detection of HER2⁺ CTCs in HER2⁻ breast cancer may predict sensitivity to anti-HER2 agents [6]. In parallel, bone-directed therapies like zoledronic acid and denosumab have been associated with reductions in CTC burden, with dynamic changes in RANK expression predicting therapeutic benefit [87, 88]. Together, these findings underscore that CTC heterogeneity not only reflects metastatic potential but also offers opportunities for monitoring treatment efficacy and guiding therapeutic stratification in bone metastasis.

Additionally, evidence suggests that CTC clusters, which are more resistant to shear stress and immune clearance, possess significantly higher metastatic potential than single CTCs. The presence of such clusters, especially those enriched with mesenchymal or stem cell markers, has been specifically associated with the early establishment of bone metastases, worse outcome and incomplete therapeutic control [15]. And the elevated metastatic competence largely stems from the synergistic effects of diverse cellular partners within heterotypic CTC clusters. Platelets rapidly coat CTCs, enhancing plasticity and vascular permeability through YAP1 and ATP–P2Y2 signaling [89], while shielding them from T and NK cell attack via the GARP–TGFβ axis and MHC class I [90]. Neutrophils, recruited through CXCL5/7 chemotaxis, form VCAM1-mediated contacts that boost proliferation, facilitate extravasation via extracellular traps or protease secretion, and provide immune protection [91]. Cancer-associated fibroblasts further promote collective invasion through E- and N-cadherin junctions, guiding migration and supporting colonization [92]. These heterotypic interactions endow CTC clusters with enhanced survival, immune evasion, and metastatic capacity. Analyzing their counts and cellular composition might potentially refine risk stratification, guide therapeutic decisions, and improve prognostic assessment in breast cancer bone metastasis. However, whether heterotypic CTC clusters confer distinct prognostic implications compared to homotypic clusters remains unclear and warrants prospective clinical validation.

CTCs have emerged as informative biomarkers in breast cancer metastasis, including bone involvement. Their dynamic nature and phenotypic plasticity indicate potential utility for early detection, prognostication, and therapeutic monitoring, although these applications require prospective validation. Preliminary studies suggest that fluctuations in CTC burden may precede radiographic evidence of progression; however, no prospective study has yet established CTCs as a stand-alone biomarker for tracking bone metastasis, highlighting the need for further validation in well-designed clinical cohorts.

## Challenges and prospects

**Fig. 3:**
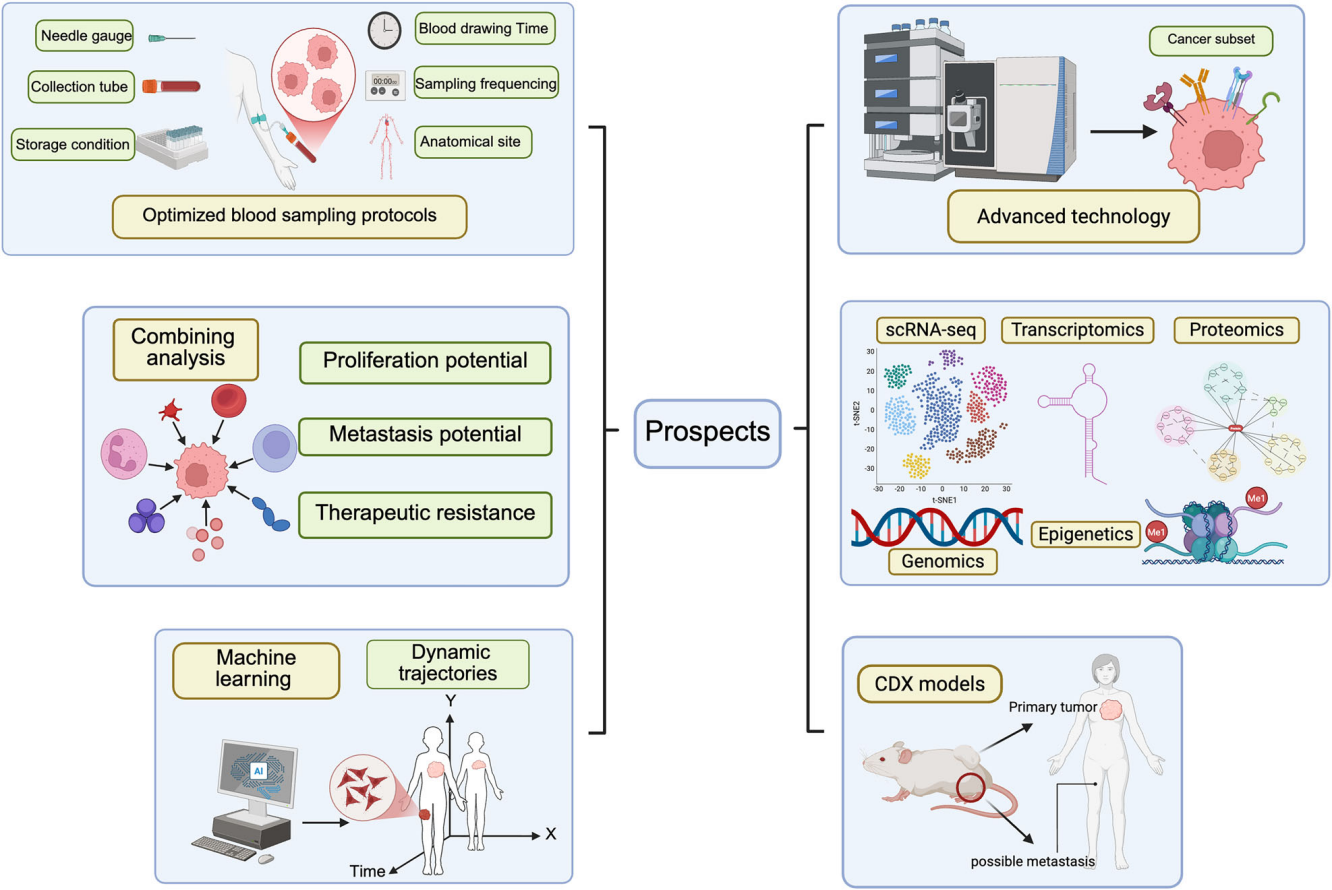
Challenges and future directions in CTC research for breast cancer. Despite being employed as outcome measures in multiple clinical trials, current CTC detection technologies still face notable limitations. Optimizing sampling strategies—such as adjusting sampling time and frequency, selecting appropriate sampling sites, and improving collection devices and storage conditions—may enhance CTC recovery rates during the collection process. Additionally, the development of enrichment methods independent of surface markers like EpCAM could significantly reduce false-negative rates. Further exploration of the clinical significance of CTCs remains critical, including integrating analyses of CTCs with other circulating cells, proteins, and cytokines; advancing single-cell multi-omics profiling; applying machine learning and artificial intelligence to construct dynamic CTC tracking maps; and utilizing CTC-derived xenograft (CDX) models to predict tumor progression and therapeutic resistance. These strategies represent important future directions for advancing CTC research in breast cancer.

Despite substantial progress, CTCs may serve as valuable biomarkers with potential applications in early detection, prognosis, and therapeutic monitoring [15]. Dynamic assessment of CTC burden has shown particular promise, as early declines in CTC counts often correlate with radiographic responses and prolonged survival, suggesting predictive value beyond conventional prognostic scores. Nevertheless, no large prospective trial has yet validated CTCs as a stand-alone diagnostic tool for the early detection of bone metastasis. At the same time, the strong link between CTCs and metastatic progression highlights opportunities to extract clinically relevant insights, particularly through multi-omics approaches, during potentially curable stages of disease. In the following section, we outline the major challenges that must be addressed and the future directions required to realize the translational potential of CTCs in oncology (as showed in Fig. 3).

Future clinical research should incorporate study designs that allow systematic evaluation of bone-specific CTC dynamics. The most appropriate settings for such investigations include: (i) high-risk early breast cancer cohorts receiving adjuvant therapy, where longitudinal CTC monitoring could facilitate early detection of occult bone dissemination; and (ii) metastatic breast cancer cohorts undergoing serial imaging and therapeutic interventions, where dynamic assessment of CTC burden and phenotypic evolution could provide insight into treatment response and progression within the bone microenvironment. Integrating CTC analysis into these clinical contexts, particularly when combined with bone-targeted imaging and molecular profiling, may offer a powerful framework to validate bone-tropic biomarkers and develop precision monitoring strategies for breast cancer bone metastasis.

Based on the physical and biological characteristics, various technologies have been developed for CTC detection (as summarized in Table 2). Despite significant progress in CTC detection technologies, several limitations continue to hinder their broader clinical application. In summary, CellSearch® provides standardized and reproducible results but is restricted to EpCAM-positive cells [93]; microfluidic platforms allow label-free enrichment with higher sensitivity but are costly and technically demanding [94]; size and property based methods are simple and non-biased but may yield lower purity. Thus, no single platform is universally optimal, and selection depends on study aims. Additionally, current technologies vary in sample volume requirements, and in some cases, large volumes are necessary to ensure sensitivity [95]. Here, we share our thoughts on the directions of future developments. To address these limitations and enhance clinical utility, several directions for future development are proposed: (1) the creation of CTC enrichment strategies that are independent of surface marker expression to minimize false negatives [96]; (2) optimization of blood sampling protocols—including time [97], sampling frequency [98], anatomical site of collection [99], needle gauge, collection tube type, and storage conditions—to reduce sample volume requirements without compromising detection efficiency; (3) the application of machine learning and artificial intelligence approaches to analyze CTC datasets, which may reduce observer and inter-laboratory variability [100]; and (4) refinement of CTC phenotyping techniques to identify highly invasive and organotropic CTC subpopulations with greater precision [101].

**Table 2:** Summary of CTC detection methods: FDA-approved and widely used research approaches.

	Method	FDA approved	Detection principle	Breast cancer application	Advantages	Limitations
Immunoaffinity-based methods	CellSearch®^a^	Yes	EpCAM-based magnetic bead enrichment + CK/CD45/DAPI immunostaining	Widely used	Clinically validated; standardized; FDA approved	EpCAM-dependent; unable to detect EMT-like CTCs; fixed cells not viable
	CELLTRACKS AUTOPREP®^a^	Yes	Automated processing platform for CellSearch	Widely used	High reproducibility; fully automated	Same as above; costly
	AdnaTest®^a^	No	Magnetic bead enrichment (EpCAM/HER2) + multiplex RT-PCR	Widely used	Enables molecular subtyping (e.g., HER2); useful for therapeutic guidance	Non-quantitative; lacks morphological assessment
	MACS® (Miltenyi Biotec)	No	Positive (EpCAM) or negative (CD45) magnetic separation	Common	Flexible enrichment strategy; viable cells	Antibody-dependent; potential for false positives/negatives
	CTC-iChip	No	Microfluidic chip + immunomagnetic leukocyte depletion	Common	Detects EpCAM-negative CTCs; viable cells for downstream analysis	Technically complex; still under research
	Epic Sciences™^a^	No	No enrichment; whole blood fixation + multiplex immunofluorescence	Widely used	Detects atypical/EMT CTCs; preserves cell morphology	Imaging is time-consuming; AI/analyst dependent
Label-free physical property-based methods	ClearCell® FX	No	Inertial microfluidics (size and deformability-based separation)	Common	Antigen-independent; captures EMT-type CTCs; viable cells	May miss small/deformable CTCs; expensive platform
	Parsortix™ (ANGLE)^a^	No	Microfluidic filtration based on cell size and deformability	Widely used	Captures EMT CTCs; allows downstream culture and analysis	Low throughput; efficiency depends on biophysical properties
	Cyttel™^a^	No	Size/deformability-based filtration + immunofluorescence confirmation	Only in China	Label-free; preserves viability; suitable for culture and multi-omics	Limited international validation; needs further standardization
	DEPArray™	No	Dielectrophoresis-based single-cell manipulation and sorting	Common	Allows single-cell recovery; suitable for genomic/proteomic analysis	High cost; low throughput; complex operation

This table summarizes major circulating tumor cell (CTC) detection platforms, categorized based on their detection principles, regulatory approval status, application in breast cancer research, and associated advantages and limitations. Methods include immunoaffinity-based and physical property-based strategies, highlighting key features such as enrichment mechanisms, cell viability preservation, and technical challenges. CellSearch® and CELLTRACKS AUTOPREP® are currently the only FDA-approved systems for clinical CTC detection, while other platforms are widely employed in academic or translational research settings.

^a^Indicates methods widely used in breast cancer research and clinical studies.

Although analyzing CTCs and their molecular markers, gene expression differences, and other factors can make great assistance, information obtained solely from CTCs still has certain limitations and cannot fully represent the patient’s condition. Therefore, combining analysis with other cells or proteins to more comprehensively assess the patient’s situation might become an important direction for the clinical application of CTCs and a research hotspot. Recent studies have shown that the combined analysis of CTCs and normal circulating cells can provide new clinical information for anti-tumor therapy. Hematological parameters such as platelet count [102], RBC count [88], and inflammatory markers [103] are significantly associated with CTC presence and phenotype in breast cancer, offering potential prognostic value and therapeutic implications. Furthermore, various serum markers, including tartrate-resistant acid phosphatase (TRACP) [104], procollagen type I N-terminal propeptide (P1NP) [105], and secreted Y-box binding protein 1 (sYB-1) [106], are associated with bone metastasis in breast cancer, with some markers showing potential for dynamic monitoring and improved diagnostic accuracy, though combined analysis with CTCs remains underexplored. And exosomes [107] and ctDNA [8] offering potential for early detection of bone metastasis through ultra-sensitive mutation analysis, is limitated due to spatial heterogeneity and short half-life, but it can be overcome by combining it with CTC analysis, enhancing diagnostic sensitivity and accuracy.

CTCs must interact with various cellular and molecular components of the bone microenvironment to establish metastatic lesions. However, large-scale characterization of CTCs has thus far been limited to basic enumeration and phenotypic profiling. Understanding their complex biological behaviors within metastatic niches—and extrapolating these findings to tumor progression in patients—requires reliable experimental models. Ideally, CTC-derived xenograft (CDX) models can recapitulate the genetic, metabolic, and behavioral features of the donor patient’s tumor, providing a platform for functional analysis and drug testing [108]. Nevertheless, CDX models, typically established in mice or other animals, differ significantly from human systems in terms of immune and metabolic context, limiting their translational relevance. Furthermore, CDX models are donor-specific and resource-intensive, making them impractical for large-scale cohort studies. To overcome these challenges, future research may benefit from the development of in vivo barcoding strategies [109] based on CTCs and their CDX models. Such approaches could enable phenotypic mapping of individual CTC subpopulations and the reconstruction of metastatic trajectories, ultimately contributing to a comprehensive atlas of breast cancer metastasis.

## Conclusion

CTCs provide a unique and dynamic window into the biology of breast cancer metastasis. Although this review primarily focuses on bone metastasis, given its clinical relevance and biological complexity, similar CTC-based monitoring strategies may also be applicable to other metastatic sites, such as the liver, lung, and brain, warranting further comparative investigation. The distinct physical and multi-omic characteristics of CTCs, together with their functional involvement in the metastatic cascade, position them as both mediators of disease progression and promising clinical biomarkers. Despite substantial advances in CTC detection and characterization, several technical and biological challenges still hinder their integration into routine clinical practice. Future research should aim to overcome these limitations to fully realize the diagnostic and therapeutic potential of CTCs, particularly within the context of breast cancer bone metastasis.

### Declaration of generative AI and AI-assisted technologies in the writing process

During the preparation of this work, LFM used ChatGPT in order to improve the readability and language of the manuscript. After using this tool, LFM reviewed and edited the content as needed and takes full responsibility for the content of the published article.
